# Pathogenic roles and diagnostic utility of interleukin-18 in autoinflammatory diseases

**DOI:** 10.3389/fimmu.2022.951535

**Published:** 2022-09-22

**Authors:** Masaki Shimizu, Syuji Takei, Masaaki Mori, Akihiro Yachie

**Affiliations:** ^1^ Department of Child Health and Development, Graduate School of Medical and Dental Sciences, Tokyo Medical and Dental University, Tokyo, Japan; ^2^ Department of Pediatrics, Field of Developmental Medicine, Graduate School of Medical and Dental Sciences, Kagoshima University, Kagoshima, Japan; ^3^ Department of Lifetime Clinical Immunology, Graduate School of Medical and Dental Sciences, Tokyo Medical and Dental University, Tokyo, Japan; ^4^ Division of Medical Safety, Kanazawa University Hospital, Kanazawa, Japan

**Keywords:** IL-18, systemic juvenile idiopathic arthritis, adult Still's disease, inflammasomes, NLRC4, pyogenic sterile arthritis, pyoderma gangrenosum, acne

## Abstract

Interleukin (IL)-18 is a pleiotropic, pro-inflammatory cytokine involved in the regulation of innate and adaptive immune responses. IL-18 has attracted increasing attention as a key mediator in autoinflammatory diseases associated with the development of macrophage activation syndrome (MAS) including systemic juvenile idiopathic arthritis and adult-onset Still’s disease. In these diseases, dysregulation of inflammasome activity and overproduction of IL-18 might be associated with the development of MAS by inducing natural killer cell dysfunction. Serum IL-18 levels are high in patients with these diseases and therefore are useful for the diagnosis and monitoring of disease activity. In contrast, a recent study revealed the overproduction of IL-18 was present in cases of autoinflammation without susceptibility to MAS such as pyogenic sterile arthritis, pyoderma gangrenosum, and acne (PAPA) syndrome. The pathogenic and causative roles of IL-18 remain unclear in these autoinflammatory diseases. Further investigations are necessary to clarify the role of IL-18 and its importance as a therapeutic target in the pathogenesis of autoinflammatory diseases.

## Introduction

Interleukin (IL)-18 is a pleiotropic, pro-inflammatory cytokine involved in the regulation of innate and adaptive immune responses (1). IL-18, originally identified as an interferon (IFN)-γ inducing factor, was isolated from the serum of mice pretreated with *Proprionibacterium acnes*, which stimulated Kupffer cells after stimulation with intraperitoneal lipopolysaccharide (2, 3). IL-18 is expressed in a wide range of cells including intestinal epithelial cells, keratinocytes, astrocytes, and endothelial cells, although the main cellular sources of IL-18 are macrophages, Kupffer cells, and dendric cells (2–7). Pro IL-18, an inactive precursor of IL-18, is cleaved by caspase-1, which is activated by the inflammasome, an intracellular multimolecular complex that plays an important role in innate immune responses as a sensor for pathogen-associated and danger-associated molecular patterns. It is then secreted as active, mature IL-18. The processing of IL-18 is mediated by the NLRP3 and the NLRC4 inflammasomes in immune cells (8, 9) and the NLRP6 and Nlrp9b inflammasomes in intestinal epithelial cells (10, 11). Secreted IL-18 binds to the IL-18 receptor, and initiates cell signaling *via* MyD88, IRAK, and TRAF6, which subsequently leads to the activation of the transcription factor NF-κB. The biological activity of IL-18 is regulated by IL-18 binding protein (IL-18BP) (12), which has a high affinity for IL-18 and is abundantly present in serum, with a greater than 20-fold molar excess compared with IL-18. IL-18BP binds to active IL-18 to form an inactive IL-18-IL-18BP complex. IL-18BP prevents active IL-18 from binding to IL-18 receptor and IL-18 function including IFN-γ production (12, 13). IL-18BP binds to IL-37 and forms an IL-18BP-IL-37-IL-18 receptor β subunit complex, inhibiting the formation of a functional receptor with an IL-18R α chain (14).

Immunological diseases can be divided into two major categories, autoimmune diseases and autoinflammatory diseases (15). Autoimmune diseases are characterized by a self-directed inflammation caused by aberrant adaptive immune cells responses leading to breaking self-tolerance and development of immune reactivity towards self-antigens. In contrast, autoinflammatory diseases are characterized by a self-directed tissue inflammation independent of adaptive immune cells abnormalities, where local factors at sites predisposed to disease lead to activation of innate immune cells. Proinflammatory cytokines derived from innate immune cells including macrophages and neutrophils play a pivotal role in the pathogenesis of autoinflammatory diseases. Among innate proinflammatory cytokines, IL-18 has attracted increasing attention as a key mediator in autoinflammatory diseases associated with the development of macrophage activation syndrome (MAS) including systemic juvenile idiopathic arthritis (16–25), adult-onset Still’s disease (26–30), XIAP deficiency (31, 32), NLRC4 gain of function (8, 33), neonatal onset of cytopenia, autoinflammation, rash, and episodes of hemophagocytic lymphohistiocytosis (NOCARH syndrome) (34) and purine nucleoside phosphorylase (PNP)-deficiency (35). Furthermore, a recent study reported the overproduction of IL-18 in cases of autoinflammation without susceptibility to MAS such as pyogenic sterile arthritis, pyoderma gangrenosum, and acne (PAPA) syndrome (36).

The aims of this review were to summarize the current evidence for IL-18 as a pathogenic mediator of autoinflammatory diseases and its potential utility as a biomarker for disease activity, severity, and prognosis.

## IL-18 biology

**Figure 1:**
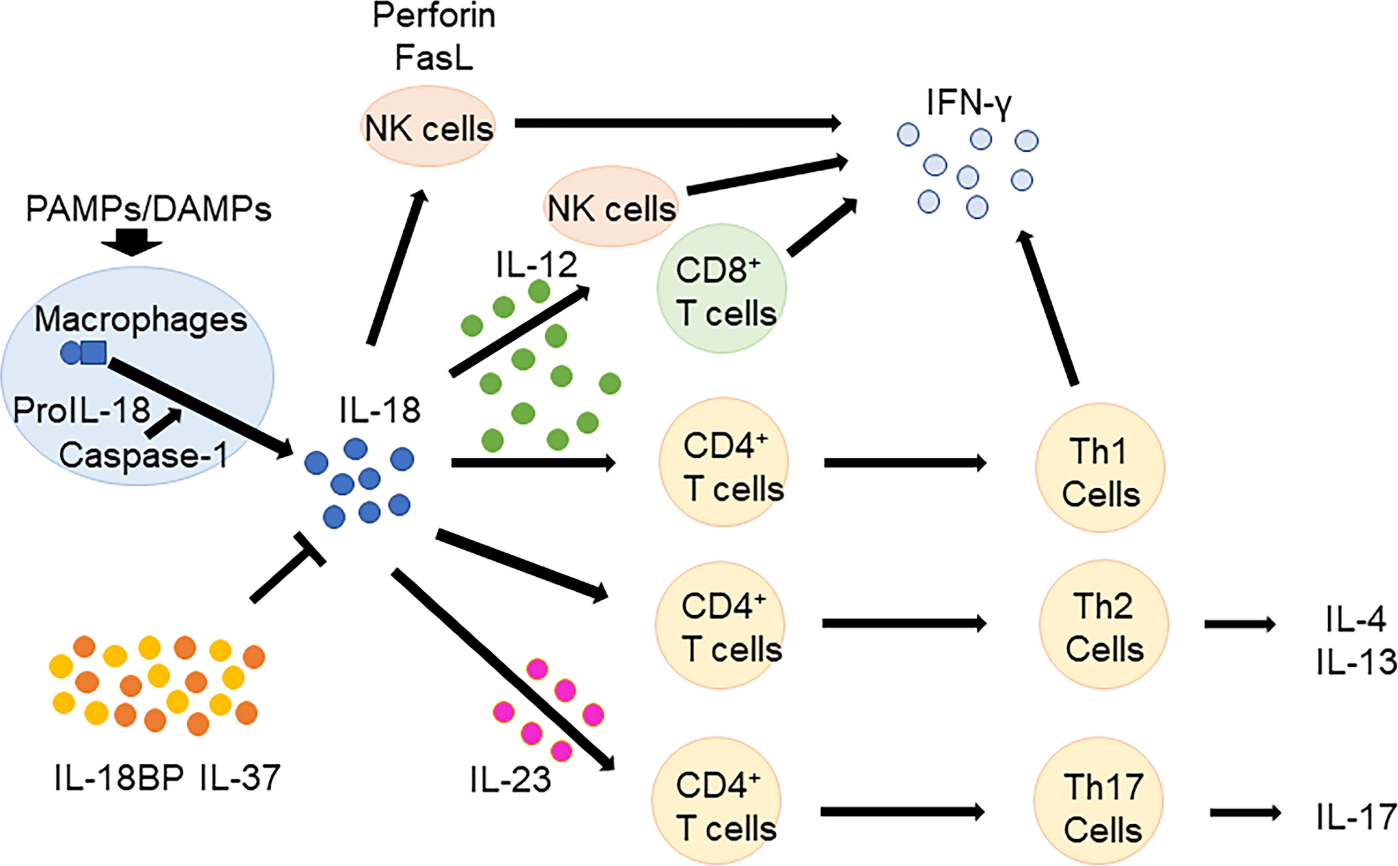
Biological functions of IL-18. In the presence of IL-12 and/or IL-15, IL-18 induces IFN-γ production in macrophages, NK cells, T cells, and B cells. In the absence of IL-12 or IL-15, IL-18 promotes the differentiation of naïve T cells to Th2 cells, which produce IL-4 and IL-13. Furthermore, IL-18 promotes Th17 cells in combination with IL-23. IL-18 BP and IL-37 regulates IL-18 function. IL, Interleukin; CD, cluster of differentiation; NK, natural killer; IFN, interferon; Th, T helper; BP, binding protein; PAMPs, pathogen associated molecular patterns; DAMPs, damage associated molecular patterns.

IL-18 has pleiotropic biological effects (**Figure 1**). In the presence of IL-12 and/or IL-15, IL-18 induces IFN-γ production in macrophages, natural killer (NK) cells, dendritic cells, T cells, and B cells (37–40). In contrast, in the absence of IL-12 or IL-15, IL-18 promotes the differentiation of naïve T cells to Th2 cells, which produce IL-4 and IL-13 (41, 42). IL-18 also induces IL-4 and IL-13 production in basophils and mast cells (43). Furthermore, IL-18 induces IL-17 production by γδ T-cells (44) and promotes Th17 cells in combination with IL-23 (1). IL-18 also plays a role in T regulatory cell (Treg) differentiation during *Helicobacter pylori* chronic infection (45). Independent of other cytokines, IL-18 induces the expression of cell adhesion molecules, nitric oxide synthesis, and chemokine production (46–48). IL-18 activates NK cells by upregulating perforin-and FasL-dependent cytotoxicity in an IFN-γ independent manner (49).

## Role of IL-18 in the pathogenesis of autoinflammatory diseases

High levels of serum IL-18 levels were reported in patients with systemic juvenile idiopathic arthritis (s-JIA) (16–25) and its adult homolog adult Still’s disease (AOSD) (26–30), as well as various other inflammatory diseases including systemic lupus erythematosus (50) and Crohn’s disease (51). s-JIA and AOSD are clinically characterized by systemic features including spiking fever, skin rash, hepato/splenomegaly, generalized lymphadenopathy, serositis, and chronic arthritis, whereas arthritis can be absent in AOSD. Laboratory findings show neutrophilia, thrombocytosis, hyperferritinemia, and increased levels of inflammation markers such as C reactive protein and serum amyloid A. Recent studies revealed proinflammatory cytokines of the innate immune system, especially, IL-1, IL-6, and IL-18 play an important role in the pathogenesis of s-JIA and AOSD (52).

IL-1 upregulates endothelial adhesion molecules and facilitates the transmigration of neutrophils and other leukocytes. IL-1 activates these cells and amplifies the inflammatory response by the activation of local fibroblasts, chondrocytes, and macrophages. IL-1 also promotes osteoclast differentiation. Furthermore, IL-1 can promote proinflammatory T cell differentiation in the direction of Th17 by the action of IL-6 and other cytokines, which might play an important role in the chronic arthritis of s-JIA and AOSD (44, 53, 54). IL-1α and IL-1β bind the same receptor, namely IL-1R1, and induce the same pro-inflammatory effects including the synthesis of cytokines, interferons and chemokines; increased expression of adhesion molecules on endothelial cells and migration of immune cells into inflamed tissues, activation of adaptive immunity such as maturation and expansion of T and B cells. However, there are distinct differences in the biology of IL-1α and IL-1β. IL-1α is constitutively expressed in various cells, and its expression can be induced by inflammatory stimuli in both hematopoietic and nonhematopoietic cells (55). Not only the cleaved form of IL-1α but also IL-1α precursor (pro-IL-1α) are biologically equally active. Inflammasomes and caspase-1 have no direct effects in cleaving pro-IL-1α. ProIL-1α without the processing of maturation is released by necrotic cells and acts as an alarmin (56). In contrast, IL-1β is absent at steady state and its expression is induced upon stimulation in innate myelomonocytic cells. Pro-IL-1β is biologically inactive and cannot work as an alarmin. The mature form of IL-1β cleaved by caspase-1 is biologically active and has a function as a soluble mediator.

IL-6 induces systemic inflammation by promoting nerve cell differentiation, the dysregulated production of pain mediators in neurons, acute phase protein production in hepatocytes, dysregulation of effector and regulatory B lymphocytes and antibody production in B cells, and dysregulation of the T cell balance. IL-6 also plays an important role in local inflammation in inflamed joints by promoting osteoclast differentiation and activation, the dysregulated production of inflammatory mediators, bone homeostasis, proliferation of fibroblasts and other cells, and matrix and bone degradation. These effects of IL-6 are closely associated with the clinical symptoms of s-JIA and AOSD including joint destruction, bone loss and growth impairment, pain, fatigue, anemia, and fever (57).

The importance of IL-1 and IL-6 in the pathogenesis of s-JIA and AOSD was proven by the dramatic effects of IL-1 inhibitors (anakinra, rilonacept, and canakinumab) and IL-6 inhibitors (tocilizumab [TCZ], sarilumab) in patients with s-JIA and AOSD (58–62). However, MAS, secondary hemophagocytic lymphohistiocytosis followed by rheumatic diseases, occurs even in patients with s-JIA and AOSD receiving IL-1 and IL-6 inhibitors (63–67).

MAS is a potentially life-threatening complication clinically characterized by sustained high fever, hepato/splenomegaly, and central nervous system dysfunction (68). Laboratory findings showed cytopenia in all three blood cell lines, hepatic dysfunction, coagulopathy, hyperferritinemia, NK cell dysfunction, and the presence of hemophagocytic macrophages in bone marrow (68). MAS is a complication in 7%–14% of children with s-JIA (69, 70) and 10%–15% of patients with AOSD (71, 72). MAS may occur subclinically in another 30%–40% of patients (73).

We reported that serum IL-18 levels were increased in patients with s-JIA and AOSD, and these were further elevated in patients with MAS (16, 17, 20, 22, 23, 26). The marked elevation of serum IL-18 levels seems to be specific to MAS with s-JIA and AOSD, and patients with secondary HLH caused by other diseases had lower serum IL-18 levels compared with sJIA related MAS (17, 22). Furthermore, we reported there were two distinct groups of patients with s-JIA/AOSD having specific clinical features based on serum IL-6 and IL-18 levels (20, 26). s-JIA/AOSD patients with an IL-6–dominant pattern (s-JIA:IL-18/IL-6 <1000, AOSD IL-18/IL-6 <5000) had more severe joint disease, whereas those with an IL-18–dominant pattern (s-JIA:IL-18/IL-6 >1000) had a more severe systemic disease and developed MAS (20, 26).

Recently, patients with gain-of-function mutations in the *NLRC4* gene have been identified (8, 33). Gain-of-function mutations in *NLRC4* caused sustained caspase-1 cleavage and the production of active IL-18 (8, 33). Patients with gain-of-function mutations in *NLRC4* can be clinically characterized by early-onset, recurrent MAS and inflammatory bowel disease, and very high IL-18 levels similar to those in patients with MAS associated with s-JIA and AOSD (8, 33). The discovery of this new disease revealed a close association between IL-18 and the development of MAS. On the other hand, some patients with gain-of-function mutations in *NLRC4* showed different clinical symptoms like cryopyrin-associated periodic syndrome not associated with MAS, despite high serum levels of IL-18 (74).

X-linked inhibitor of apoptosis (XIAP) deficiency with *XIAP/BIRC4* gene mutations is a primary immune deficiency clinically characterized by HLH, inflammatory bowel disease, and splenomegaly (75). XIAP belongs to the inhibitor of apoptosis family of proteins and inhibits caspases 3, 7, and 9, and has an antiapoptotic role (76–78). XIAP regulates multiple immune pathways including NOD1 and NOD2 signaling, Dectin1 signaling, TNF-receptor signaling, and the NLRP3 inflammasome. Therefore, the loss of XIAP induces the dysregulation of inflammasome activity and overproduction of inflammasome-activated cytokines (31). We previously reported serum IL-18 levels were highly elevated in XIAP deficient patients with HLH and that this elevation was sustained in the inactive phase after recovery from HLH (31). These findings indicate that NLRC4 and XIAP play an important role in regulating IL-18, and that the dysregulation of inflammasome activity and overproduction of IL-18 are closely associated with the development of MAS.

NK cell dysfunction is a characteristic finding of HLH (79) and was observed in patients with s-JIA (80–84). IL-18 increases NK cell activity. However, exposure to high levels of IL-18 can induce NK cell death (81–84). A previous report showed that NK cell dysfunction in s-JIA was closely associated with a defect in IL-18 receptor β phosphorylation (81). Furthermore, Ohya et al. recently demonstrated the impaired phosphorylation of MAPK p38 and NFκB p65 in NK cells following rIL-18 stimulation in patients with active s-JIA (83). These findings indicate that IL-18 exposure might induce NK cell dysfunction and reduce the number of NK cells. We previously reported infants born to mothers with AOSD can develop MAS and show extremely elevated levels of IL-18 (85). Furthermore, infants born to mothers with AOSD showed increased serum IL-18 levels, transmitted from the mother to the infant, as well as the transient impairment of NK cell functions (86). Furthermore, we reported that NK cell activation by exogenous IL-18 stimulation was impaired in patients with active s-JIA and high serum levels of IL-18 (82). Interestingly, NK cell activation was restored by IL-18 stimulation in patients with s-JIA after treatment began and serum IL-18 levels had decreased. Furthermore, Ohya et al. reported that the impaired phosphorylation of MAPK p38 and NF-κB p65 in NK cells following rIL-18 stimulation was recovered to normal levels with improvements in the patients’ clinical condition after treatment began (83). These findings indicate NK cell exhaustion and secondary transient NK cell dysfunction induced by exposure to high serum IL-18 levels may be closely associated with MAS development in s-JIA. However, NK cells were normal in number and function in patients with gain-of-function mutations in *NLRC4* and XIAP deficiency. These findings indicate IL-18 overproduction might be associated with MAS development by inducing other mechanisms besides NK cell dysfunctions.

Recently, a case of IL-18BP deficiency with fulminant hepatitis caused by hepatitis A virus infection was reported (87). Furthermore, IL-18BPKO mice receiving repeated TLR9 stimulation developed MAS with severe hepatic inflammatory damage, which was reversed by the treatment of IL-18BP (88). These findings indicate that IL-18 might be closely associated with severe liver injury in s-JIA/AOSD and related MAS. In patients with s-JIA and AOSD, IL-18 expression was increased in *reticuloendothelial cells in the* liver, lymph nodes, and inflamed tissues (89–91). A previous report showed IL-17-expressing γ/δ T cells might play an important role in the chronic arthritis of s-JIA (44). Furthermore, a recent report showed a shift in Th17 responses from Tregs in acute disease to effector T cells in chronic disease in patients with s-JIA (54). IL-18 induced IL-17 production by γδ T-cells (44) and promoted Th17 in combination with IL-23 (1, 42). s-JIA patients with chronic arthritis had sustained elevated serum IL-18 levels (23). Taken together, these findings indicate IL-18 might play a role in the chronic arthritis of s-JIA through the persistent activation of IL-17 responses.

**Figure 2:**
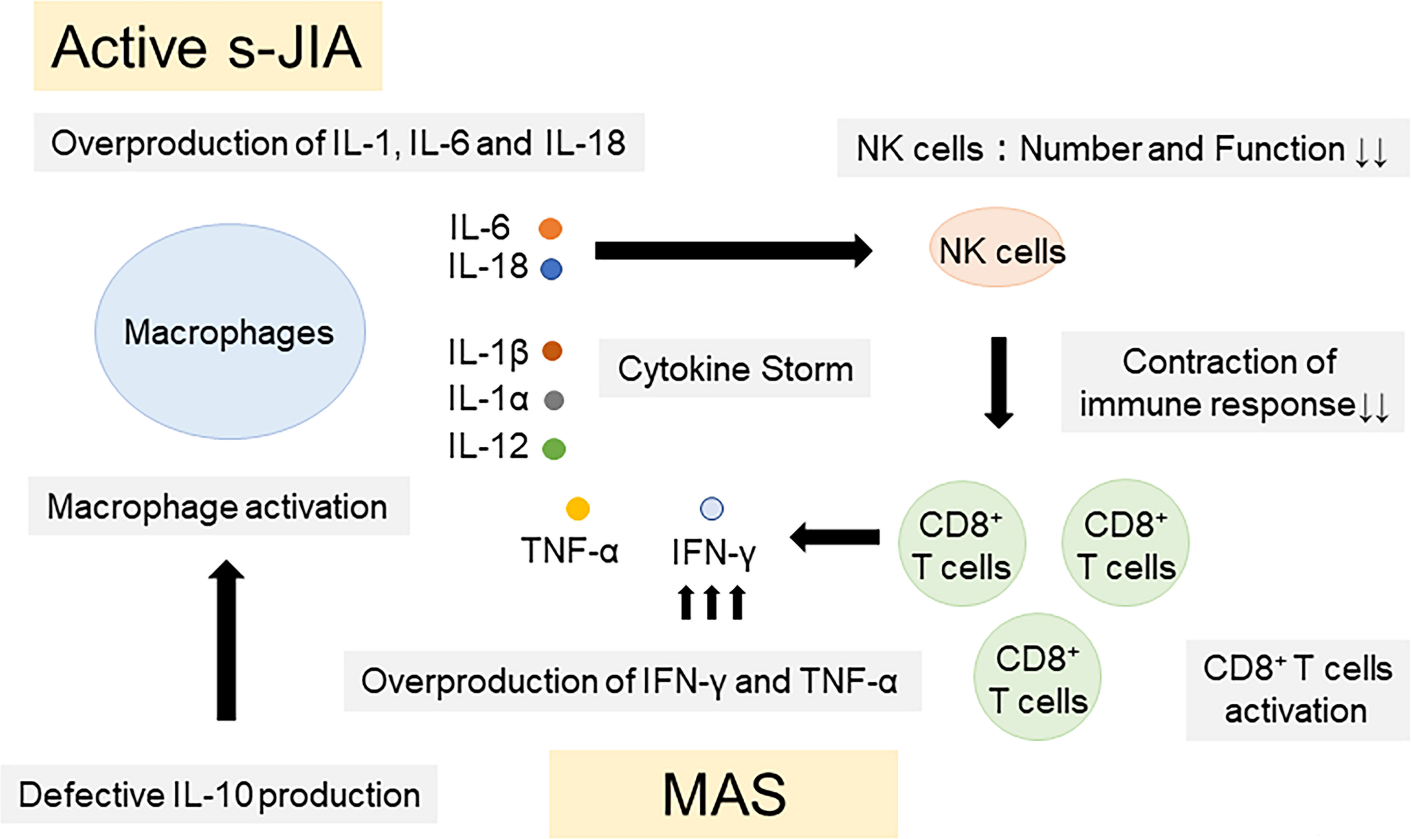
Pathogenic mechanisms of IL-18 in the pathogenesis of s-JIA and MAS. Overproduction of IL-1, IL-6 and IL-18 is a hallmark of active phase of s-JIA. *In vivo* exposure highly elevated IL-6 and IL-18 levels and induced NK cell dysfunction and decreased the number of cells. Defective NK cells are unable to contract immune responses, leading to massive expansion and activation of CD8+ T cells and overproduction of IFN-γ and TNF-α. Furthermore, inadequate production of IL-10, a regulatory cytokine to counter-regulate IFN-γ, might be related to the development of MAS. IL, Interleukin; TNF, tumor necrosis factor; IFN, Interferon; CD, cluster of differentiation; NK, natural killer; s-JIA, systemic juvenile idiopathic arthritis; MAS, macrophage activation syndrome.

Pathogenic mechanisms of IL-18 in the pathogenesis of s-JIA and MAS were summarized in **Figure 2**. Overproduction of IL-1, IL-6 and IL-18 is a hallmark of active phase of s-JIA. *In vivo* exposure highly elevated IL-6 and IL-18 levels and induced NK cell dysfunction and decreased the number of cells. Defective NK cells are unable to contract immune responses, leading to massive expansion and activation of CD8+ T cells and overproduction of IFN-γ. Furthermore, inadequate production of IL-10, a regulatory cytokine to counter-regulate IFN-γ, might be related to the development of MAS. IFN-γ plays a pivotal role in the development of MAS. IFN-γ binds to the receptors on macrophages and activates them. Activated macrophages release even more proinflammatory cytokines including TNF-α, IL-1 and IL-6, leading to the cytokine storm. High serum IFN-γ levels are found in patients with primary HLH and secondary HLH including MAS (92–96). Furthermore, clinical symptoms of HLH have been shown to be inhibited by anti-IFN-γ antibody treatments in patents with HLH as well as animal models of HLH (97–104).

Recently, a novel hematological and autoinflammatory syndrome clinically characterized by neonatal-onset cytopenia with dyshematopoiesis, autoinflammation, rash and HLH (NOCARH syndrome) was identified (34). The pathogenic variant, p.R186C at the C-terminus of cell division control protein 42 (CDC42) was specifically associated with this syndrome (34). Patients with this variant in CDC42 showed sustained extreme elevation of serum IL-18 levels, likely predisposing to development of MAS (34, 105). More recently, additional 2 different pathogenic variants at the C-terminus of CDC42, pC188Y and p*192Cext*24 were proposed to cause IL-1 blocking sensitive autoinflammation (105). Although CDC42 mutations had been associated with neurodevelopmental diseases (106–110), variants at the CDC42 C-terminus can affect the localization and function of CDC42 protein, and cause autoinflammation. Arg186 and Arg 187 in C-terminal diarginine motif of CDC42 is important for the binding of CDC42 to phosphatidylinositol 4,5-bisphosphonate (PIP2), whose interaction of CDC42 is critical in mediating the PIP2-induced actin assembly (111). Aberrant actin depolymerlization can activate the inflammasome and modulate innate immune functions (112, 113). The functional effect of actin polymerization defects caused by variants at the CDC42 C-terminus might lead to autoinflammation.

Purine nucleoside phosphorylase (PNP)-deficiency is a primary immune deficiency with *PNP* gene mutation, clinically characterized by a progressive combined immunodeficiency, neurologic symptoms including developmental delay, spasticity, ataxia, and pyramidal signs, and autoimmune manifestations including hemolytic anemia (114–118). PNP is one of the enzymes involved in purine salvage. Thus, PNP deficiency leads to intracellular accumulation of deoxyguanosine triphostate (dGTP) which induces toxic effects for neurons and T lymphocytes (114–117). Recently, a patient with PNP-deficiency whose clinical manifestation was highly suggestive for sJIA complicated with MAS has been reported (35). High serum IL-18 levels have been described in patients with PNP-deficiency (35, 118). PNP-deficiency is associated with overproduction of IL-18 and they are predisposed to develop MAS, although it remains unclear how PNP-deficiency affects overproduction of IL-18 and induces MAS development.

PAPA syndrome caused by *PSTPIP1* gene mutations is clinically characterized by pyogenic sterile arthritis, pyoderma gangrenosum, and acne (36). Mutations of the *PSTPIP1* gene induce increased binding to pyrin and the activation of NLRP3 inflammasomes (119, 120). PSTPIP1 interacts with the actin cytoskeleton by regulating the Wiskott-Aldrich Syndrome protein (119, 120). Thus, mutations of the *PSTPIP1* gene can alter innate immune cell motility. Recently, Stones et al. reported serum IL-18 levels were highly increased in patients with PAPA syndrome similar to those in patients with MAS associated with s-JIA and AOSD (36). Serum IL-18 levels were significantly elevated in some patients with familial Mediterranean fever (FMF), although they were not significantly elevated in most patients with FMF (36, 121). A recent report revealed FMF patients carrying M694I and E148Q mutations in the *MEFV* gene had significantly higher levels of serum IL-18 compared with those carrying M694I, but not E148Q, and those carrying E148Q, but not M694I (121). Although the specific mechanism driving IL-18 through the pyrin inflammasome remains unknown, the dysregulation of interactions between pyrin and PSTPIP1 caused by *MEFV* and *pstpip1* gene mutations might induce the overproduction of IL-18.

MAS is not a complication in patients with PAPA syndrome. Therefore, IL-18 may be elevated in PAPA syndrome without increasing the risk of MAS development. The possible reasons why IL-18 significantly elevated in PAPA syndrome without increasing the risk of MAS are 1) high enough IL-18BP level in serum, 2) differences in the inflammatory milieu of s-JIA and PAPA syndrome, and 3) differences in the cellular source of IL-18. Furthermore, elevated levels of IL-18 alone might not be able to induce MAS development. IL-18 has been studied in murine models of HLH/MAS and IL-18 blocking could not improve their survivals (88). IL-10 counter-regulates IFN-γ. In a mouse model of MAS, IL-10 blocking exacerbated the disease activity of MAS (122). A previous study revealed IL-10 production in B lymphocytes was impaired, and plasma IL-10 levels plasma were not relatively elevated compared with other proinflammatory cytokines in patients with s-JIA (123). These findings indicate that other pathogenic pathways including aberrant production of IL-10 production might contribute to the pathogenesis of MAS in addition to IL-18 overproduction induced autoinflammation.

To summarize, autoinflammatory diseases characterized by high levels of IL-18 were shown in **Table 1**.

**Table 1:** Autoinflammatory diseases characterized by high levels of IL-18.

Diseases	s-JIA	AOSD	NLRC4GOF	XIAP deficiency	NOCARH syndrome	PNP deficiency	PAPA syndrome	FMF
Causal gene	NA	NA	*NLRC4*	*XIAP*	*CDC42* (c-terminus)	*PNP*	*PSPPIP1*	*MEFV*
MAS susceptivity	+	+	+	+	+	+	–	–
Clinical manifestations	Fever	Fever	Fever	Immunodeficiency	Fever	Immunodeficiency	pyogenic sterile arthritis	Fever
	Rash	Rash	Rash	Inflammatory bowel disease	Cytopenia with dyshematopoiesis	Neurologic symptoms	pyoderma gangrenosum	Rash
	Lymphadenopathy	Lymphadenopathy	Arthralgia	Splenomegaly	Rash	Autoimmune manifestations	acne	Serositis
	Hepatomegaly	Hepatomegaly	Inflammatory bowel disease					
	Splenomegaly	Splenomegaly						
	Serositis	Serositis						
		Pharyngeal pain						

## Clinical application of serum IL-18 levels as a biomarker for autoinflammatory diseases

Serum IL-18 levels are significantly and highly increased in active s-JIA (16–26), which can be useful for the differentiation of s-JIA from other inflammatory diseases (17, 23). We compared serum IL-18 levels in s-JIA and other inflammatory diseases (23) and found they were significantly elevated in patients with s-JIA compared with Kawasaki disease (KD), TRAPS, other subtypes of JIA, SLE, Juvenile dermatomyositis (JDM), and leukemia. The cut-off values of serum IL-18 levels for the differentiation of s-JIA from KD, FMF, TRAPS, other subtypes of JIA, SLE, JDM, and leukemia were 4560, 4800, 1685, 1728, 2400, 2125, and 2240 pg/mL, respectively. The cut-off value of serum IL-18 levels for the differentiation s-JIA from all other diseases was 4800 pg/ml.

Serum IL-18 levels are closely correlated with the disease course of s-JIA and are useful as a diagnostic laboratory criterion for remission in s-JIA. There are three types of disease course in s-JIA: 1) monocyclic course; no disease flare occurs; 2) chronic course; disease flare frequently occurs with steroid withdrawal; and 3) polycyclic pattern; disease flare repeatedly occurs after remission is achieved in each flare. We longitudinally measured serum IL-18 levels in s-JIA patients from the active phase through to the inactive phase or remission (23). In patients with a monocyclic course, serum IL-18 levels smoothly decreased to <1000 pg/mL in the inactive phase and sustained low levels in the remission phase. In contrast, in patients with a chronic pattern, serum IL-18 levels were sustained at >1000 pg/mL, even in the inactive phase. In patients with a polycyclic pattern, serum IL-18 levels were elevated at >1000 pg/mL during disease flares, although these levels were normalized in the inactive phase. The cut-off value of serum IL-18 levels for the diagnosis of remission in s-JIA was 595 pg/mL. These results indicate that the monitoring of serum IL-18 levels is useful to predict the disease course and assess remission in s-JIA.

Previous reports showed that TCZ could mask the clinical features and laboratory findings of patients with s-JIA (64–67). We compared serum IL-18 levels between s-JIA patients receiving TCZ and not receiving TCZ (23, 64) and found no statistically significant differences between the groups. Thus, the monitoring of serum IL-18 levels is useful for the assessment of disease activity in s-JIA, and in patients with s-JIA receiving TCZ.

Serum IL-18 levels are also elevated in patients with s-JIA–associated MAS (16, 17, 20–26). We compared serum IL-18 levels in s-JIA patients with active disease who later developed MAS and those who did not (20). Serum IL-18 levels in the former group were significantly higher than in the latter group in the active phase prior to the development of MAS as well as in the MAS phase. In contrast, there was no significant difference in serum IL-18 levels in the former group whether measured before or during MAS. The cut-off value for serum IL-18 levels to predict MAS development was 47,750 pg/ml. We compared the accuracy of serum biomarkers including neopterin, IL-18, CXCL9, and soluble tumor necrosis factor receptor type I (sTNF-RI) and II for the diagnosis of MAS complicating s-JIA (124). Serum neopterin levels might be the most useful marker to diagnose the transition to MAS from active-phase s-JIA.

In addition to serum total IL-18 levels, recent reports revealed IL-18BP-unbound, bioactive, and free IL-18 levels in serum were significantly elevated in patients with s-JIA and AOSD (24, 25, 29). Serum free IL-18 levels were significantly correlated with the disease activity of s-JIA and AOSD (24, 25, 29). Furthermore, these levels increased further in MAS associated s-JIA/AOSD (24, 25, 29). Most patients with s-JIA showed chronically elevated total serum IL-18 levels (24, 25). In contrast, elevation of free IL-18 in serum was largely restricted to s-JIA patients with MAS (25). Furthermore, serum free IL-18 levels reflected the disease activity of MAS (25). From these findings, serum free IL-18 levels might be useful as a biomarker of disease activity of MAS compared to total IL-18 levels.

Serum IL-18 levels are also useful for the prediction of treatment outcomes in patients with s-JIA treated with canakinumab (CAN) in combination with serum interferon (IFN)-γ levels and chemokine (C-X-C motif) ligand 9 (CXCL9) levels (21). Recently, Hinze et al. reported responders to canakinumab had higher serum IL-18 and IFN-γ levels and lower CXCL9 levels at baseline; that is, higher IL-18:CXCL9 and IFN-γ:CXCL9 ratios at baseline were associated with a better clinical response to CAN treatment in s-JIA (21). These ratios might have significant accuracy for the prediction of treatment responses.

We compared serum cytokine profiles and kinetics in patients with AOSD with those in patients with s-JIA (26). The cytokine patterns associated with s-JIA and AOSD shared common features including a predominant increase in IL-18. Interestingly, as well as s-JIA, patients with AOSD were classified into two subgroups based on serum IL-6 and IL-18 levels. The number of patients with arthritis was significantly higher in the IL-6-dominant group. These findings support the hypothesis that s-JIA and AOSD share a disease category. Serum IL-18 levels are significantly and highly increased in AOSD (26–30) and these levels clearly reflect the disease activity of AOSD. The cut-off value of serum IL-18 levels for the differentiation of AOSD from other febrile diseases was 5,000pg/mL (28). However, it is still unclear whether serum IL-18 levels are closely correlated with the disease course of AOSD, and whether they are also useful as a diagnostic laboratory criterion for remission in AOSD. Further larger studies may help to define the true diagnostic value of IL-18 as a biomarker of AOSD.

Serum IL-18 levels are highly increased in patients with PAPA syndrome (36). In contrast to s-JIA and AOSD, serum IL-18 levels did not correlate with clinical symptoms and acute phase reactants in patients with PAPA syndrome (36). However, there were clear differences in serum IL-18 levels between PSTPIP1 mutation–positive patients and PSTPIP1 mutation–negative patients (36). These findings indicate that the elevation of IL-18 correlates with PSTPIP1 mutations rather than clinical features. From these results, serum IL-18 might be useful to distinguish between patients carrying PSTPIP1 mutations from patients with suspicious clinical findings regardless of its pathogenic role.

## IL-18 as a therapeutic target

Free IL-18 might be a therapeutic target in s-JIA and AOSD. Tadekinig alfa, human recombinant IL-18BP, was effective in patients with MAS associated s-JIA (125), XIAP deficiency (32), and NLRC4 gain-of-function (33). Furthermore, a case report of one patient with AOSD treated with tadekinig alfa showed serum free IL-18 levels were undetectable 2 h after the administration of IL-18BP, even though the levels were high before the subcutaneous injection (126). Furthermore, serum free IL-18 levels remained low during disease remission under tadekinig alfa therapy, and then increased again after tadekinig alfa was discontinued, in parallel to relapse phases. A phase II clinical trial of tadekinig alfa in patients with refractory AOSD (127) reported about half of the patients had a normal body temperature with CRP levels decreased to 50% of their baseline levels or <5 mg/L 3 weeks after starting tadekinig alfa. Further larger studies including randomized control studies are required to confirm the clinical effect of tadekinig alfa on IL-18 driven diseases such as s-JIA and AOSD.

## Conclusions

IL-18 plays an important role in autoinflammatory diseases, in particular diseases associated with MAS including s-JIA, AOSD, XIAP deficiency, and NLRC4 gain-of-function. IL-18 inhibition is likely to be effective for these diseases. In contrast, the overproduction of IL-18 was observed in PAPA syndrome without susceptibility to MAS. The pathogenic and causative roles of IL-18 remain unclear in each disease. Further investigations are necessary to clarify the role of IL-18 and its importance as a therapeutic target in the pathogenesis of autoinflammatory diseases.

## Author contributions

MS drafted the paper; ST, MM, and AY supervised and reviewed the paper. All authors read and approved the final version of the manuscript.

## Funding

This work was supported by the Japan Society for the Promotion of Science (JAPS) KAKENHI (18K07786).

## Acknowledgments

We thank Harumi Matsukawa for technical assistance.

## Conflict of interest

Tokyo Medical and Dental University received unrestricted research grants for the salary of MM from Abbvie Japan, Asahi-Kasei, Ayumi Pharmaceutical Corporation, Chugai Pharmaceutical Co., Ltd., CSL Behring K.K., Japan Blood Products Organization, Nippon Kayaku and UCB Japan Co. Ltd., while TMDU paid the salary of MM.

The remaining authors declare that the research was conducted in the absence of any commercial or financial relationships that could be construed as a potential conflict of interest.

## Publisher’s note

All claims expressed in this article are solely those of the authors and do not necessarily represent those of their affiliated organizations, or those of the publisher, the editors and the reviewers. Any product that may be evaluated in this article, or claim that may be made by its manufacturer, is not guaranteed or endorsed by the publisher.
